# Supplementary material for: Emergence of flat bands and ferromagnetic fluctuations via orbital-selective electron correlations in Mn-based kagome metal
Source: Nat Commun. 2024 Jun 25;15:5376. doi: 10.1038/s41467-024-49674-3 (PMC11199626; doi:10.1038/s41467-024-49674-3)
Supplement: Supplementary file 1 — Supplementary Information [file 41467_2024_49674_MOESM1_ESM.pdf]

Supplementary Information for "Emergence of flat bands and ferromagnetic fluctuations via orbital-selective electron correlations in Mn-based kagome metal"

Subhasis Samanta,[1,2] Hwiwoo Park,[3] Chanhyeon Lee,[4] Sungmin Jeon,[3] Hengbo Cui,[5] Yong-Xin Yao,[6,7] Jungseek Hwang,[3,*] Kwang-Yong Choi,[3,†] and Heung-Sik Kim[1,‡]

[1]*Department of Semiconductor Physics and Institute of Quantum Convergence Technology, Kangwon National University, Chuncheon 24341, Republic of Korea*

[2]*Center for Extreme Quantum Matter and Functionality, Sungkyunkwan University, Suwon 16419 Republic of Korea*

[3]*Department of Physics, Sungkyunkwan University, Suwon 16419, Republic of Korea*

[4]*Department of Physics, Chung-Ang University, Seoul 06974, Republic of Korea*

[5]*Department of Physics and Astronomy and Institute of Applied Physics, Seoul National University, Seoul 151-747, Republic of Korea*

[6]*Ames National Laboratory, U.S. Department of Energy, Ames, Iowa 50011, USA*

[7]*Department of Physics and Astronomy, Iowa State University, Ames, Iowa 50011, USA*

[*] jungseek@skku.edu

[†] choisky99@skku.edu

[‡] heungsikim@kangwon.ac.kr

Supplementary Note 1. Experimental details

A. Sample growth, transport and magnetic characterizations

Single crystals of SMAS were grown by a self-flux method. The Sc, Mn, Al, and Si elements with a molar ratio 3:3:30:5 were ground and loaded into an alumina crucible. Then, the crucible was inserted inside a quartz tube and sealed under high-purity argon. The tube was heated to 1150 °C, kept at that temperature for 24 h, and subsequently cooled to 750 °C for 200 h. The single crystals were isolated by centrifugation. The magnetic susceptibility and magnetization were measured with a superconducting quantum interference device vibrating sample magnetometer (SQUID VSM) (Quantum Design, USA) in the temperature range of 1.8-300 K and the field range of $\mu_0 H$ = 0-7 T. Electrical resistivity measurements were carried out with a conventional four-point schema using a Quantum Design physical property measurement system (PPMS).

B. Nuclear magnetic resonance measurement

^{27}Al (gyromagnetic ratio γ_N = 11.0943 MHz/T) NMR experiments were conducted using a home-made NMR spectrometer (MagRes) and an Oxford Teslatron PT superconducting magnet in the temperature range of 2-150 K. To record ^{125}Al NMR spectra, we adopted a standard Hahn-echo (spin-echo) sequence with a $\pi/2$ pulse length of 2 μs. In the process of tracking all peaks, we varied a resonance frequency at a fixed magnetic field of $\mu_0 H$ = 6 T. The spin-lattice ($1/T_1$) and spin-spin relaxation rates ($1/T_2$) were measured with the standard saturation recovery and the Hahn echo method with twenty saturation pulse train.

C. Optical spectroscopy

We measured the reflectance of SMAS in a wide spectral range from 40 to 35000 cm^{-1} at various selected temperatures between 8 and 300 K using a commercial spectrometer Vertex 80v (Bruker, Germany) and a continuous liquid helium flow cryostat. To obtain accurate reflectance spectra, we used an *in-situ* metal evaporation method [1]. In this metal evaporation method, Au is used for far and mid-infrared regions, and Al is used for near-infrared and visible regions. The measured reflectance spectra at various temperatures are shown in Supplementary Fig. 1. We observed that the overall level of the reflectance is quite high above 40% up to 35000 cm^{-1} and a sharp plasma edge is located near 1500 cm^{-1}. In the inset, we display the reflectance at 100 cm^{-1} as a function of temperature, where we observe non-monotonic temperature-dependent behavior. There is a broad peak near 30 K, which is similar temperature-dependent behavior of the measured

DC resistivity.

Supplementary Note 2. Computational details

A. Density functional theory

Initial first-principles electronic structure calculations for the preparation of DFT+DMFT runs were carried out by employing WIEN2K code, which uses a full-potential linearized augmented plane-wave basis set combined with local orbital bases [2]. A non-shifted 10×10×8 k-grid was chosen to sample the hexagonal Brillouin zone. The muffin-tin radii of Sc, Mn, Al, and Si were set to 2.5, 2.5, 1.94, and 2.21 a.u., respectively. The LAPW basis included $3d$, $4s$ for Sc and Mn; $3s$, $3p$ for Al and Si elements. For self-consistent electronic cycles, the RK_{max} was taken to 9, yielding 5162 bases for each k-point.

Additionally, DFT+U calculations were performed using the Vienna *ab-initio* Simulation Package (VASP). 400 eV of plane-wave energy cutoff and a Gamma-centered 10×10×8 k-grid was used. For the treatment of the on-site Coulomb repulsion we employed a rotationally-invariant Dudarev scheme [3] with effective Coulomb repulsion parameter U (U_{eff}=0, 4, 8 eV) on top of a revised version of the Perdew-Burke-Ernzerhof exchange-correlation functional for solids (PBEsol) [4].

B. Dynamical mean-field theory

LDA+DMFT calculations were performed employing embedded DMFT code [5, 6], interfaced with the full potential based WIEN2K package [2]. A similar size of k-grid 10×10×8, as used in DFT calculations, was employed and RK_{max} was set to 9. The quantum impurity problem was solved in the correlated subspace of Mn d-orbitals, using hybridization-expansion continuous-time quantum Monte Carlo (CT-HYB) method [7, 8]. For each charge self-consistent DMFT iteration, 192×10^9 Monte Carlo steps were performed. The DMFT calculations were carried out for a wide range of temperature 116-2000 K. For each temperature, 50 DMFT charge cycles were run. The calculations were performed using both Ising as well as rotationally invariant full Coulomb interactions. Four different values of on-site Coulomb (U= 4, 6, 8, 10 eV) and two different choices of Hund's (J_{H} = 0.8, 1.0 eV) parameters were taken in the calculations. Most of the calculations results were obtained using (U, J_{H})=(8, 0.8) eV with full Coulomb interaction.

C. Rotationally-invariant Slave-Boson mean-field theory calculations

For our DFT+RISB calculations, we employed CYGUTZ (https://cygutz.readthedocs.io/) package

in combination with WIEN2K [9, 10]. $RK_{max} = 9.0$ was employed, and for a better convergence, a non-shifted k-grid of up to $17 \times 17 \times 14$ was used. Mn d-orbital was set to be the correlated active subspace.

Supplementary Note 3. Optical conductivity

The optical conductivity was obtained from the measured reflectance (see Supplementary Fig. 1) by using the Kramers-Kronig analysis [11, 12]. To perform the Kramers-Kronig analysis, the reflectance spectrum in a finite spectral range must be extrapolated to zero and infinity. For extrapolation to zero frequency, we used the Hagen-Rubens relation, i.e., $1 - R(\omega) \propto \sqrt{\omega}$. For extrapolation to infinity, we used $R(\omega) \propto \omega^{-1}$ from the highest data point to 10^6 cm^{-1}, and then above 10^6 cm^{-1}, we assumed the free-electron behavior, *i.e.*, $R(\omega) \propto \omega^{-4}$. The optical conductivities at various temperatures obtained using the Kramers-Kronig analysis are shown in Fig. 7a in the main text. There are many sharp infrared-active phonon absorption peaks below 500 cm^{-1}. We obtained the DC conductivity from extrapolation to zero frequency of the optical conductivity. In the inset, the DC resistivity obtained using the extrapolation and the measured DC one are shown. They are consistent with each other.

Now, to obtain information on the correlations between charge carriers, we used the extended Drude model formalism. In the extended Drude model, the complex optical conductivity can be expressed as follows [13, 14].

$$\tilde{\sigma}(\omega) = i\frac{\Omega_p^2}{4\pi}\frac{1}{\omega + \left[-2\tilde{\Sigma}^{\rm op}(\omega)\right]}, \tag{1}$$

where Ω_p is the plasma frequency of itinerant charge carriers and $-2\tilde{\Sigma}^{\rm op}(\omega)$ is the complex optical self-energy, which may carry information on the correlations between itinerant charge carriers. The imaginary part, $-2\Sigma_2^{\rm op}(\omega)$ is the same as the optical scattering rate $(1/\tau^{\rm op}(\omega))$ and the corresponding real part is associated with the optical effective mass $(m^*_{\rm op}(\omega)/m_{\rm b})$, *i.e.*, $-2\Sigma_1^{\rm op}(\omega) = \left(\frac{m^*_{\rm op}(\omega)}{m_{\rm b}} - 1\right)\omega$, where $m_{\rm b}$ is the band mass. We obtained the plasma frequency by excluding the interband transitions above 1500 cm^{-1}. The resulting plasma frequency is 14890 cm^{-1}. Here, we first removed the interband transitions from the optical conductivity and then obtained the optical scattering rates and effective masses using the extended Drude formalism. The optical scattering rates and effective masses $\left(\frac{m^*_{\rm op}(\omega)}{m_{\rm b}}\right)$ at various temperatures are shown in Supplementary Fig. 2. The value of the optical effective mass at zero frequency is the effective mass with respect

to the band mass, *i.e.*, $\frac{m^*_{\mathrm{op}}(0)}{m_{\mathrm{b}}} = \frac{m^*}{m_{\mathrm{b}}}$. The effective mass is roughly in a range from 2.8 to 3.8. It is worth noting that these values are dependent on the plasma frequency.

Supplementary Note 4. ^{27}Al nuclear magnetic resonance results

Supplementary Fig. 3a shows the thermal evolution of ^{27}Al NMR spectra of SMAS. We observe five NMR lines, which stem from the quadrupole interaction of the $I = 5/2$ nonspherical nucleus with its electronic surroundings. Supplementary Figs. 3b plots the Knight shift K versus $\chi(T)$ for the central line P_3. Here, $K(T)$ is defined by $K(\mathrm{ppm}) = (\nu_{\mathrm{P3}} - \nu_{\mathrm{Larmor}})/\nu_{\mathrm{Larmor}}$, where ν_{Larmor} is the Larmor frequency corresponding to an external field of $\mu_0 H = 6$ T. Using the Clogston-Jaccarino K-χ plot, we estimate the hyperfine interaction $A = 6.57\mathrm{mT}/\mu_{\mathrm{B}}$ between the ^{27}Al nuclear spins and the Mn electron spins. Supplementary Figs. 3c, d exhibit the temperature dependence of the nuclear spin-spin relaxation rate $1/T_2$ and the nuclear spin-lattice relaxation rate $1/T_1$, respectively. As the temperature is lowered from $T = 120$ K, $1/T_2$ decreases gradually and then, forms a minimum at 30 K, and finally shows a steep increase towards $T = 0$ K. Noteworthy is that $\rho(T)$ displays the minimum at the same temperature, implying that the $1/T_2$ and $\rho(T)$ anomalies below 30 K are due to the buildup of ferromagnetic correlations.

The temperature dependence of $1/T_1$ follows the so-called Korringa law [15] in the temperature range investigated with $T_1T = 44.88 \pm 0.24$ s·K for $T < 40$ K and $T_1T = 40.11 \pm 0.19$ s·K for $T > 40$ K. Using the value for the Knight shift and $S = (\gamma_e/\gamma_n)^2(h/8\pi^2 k_B) = 3.89 \times 10^{-6}$ s·K for ^{27}Al, we evaluate the Korringa ratio $R = K^2 T_1 T/S \approx 0.05$ at $T = 2$ K, much smaller than the unity. This indicates that $1/T_1$ is not dominated by a scattering mechanism with s-type conduction electrons at the Fermi surface. Further, the small change of the Korringa ratio through 40 K suggests the alteration of a scattering mechanism.

Supplementary Note 5. DFT+U calculation results

Supplementary Fig. 4 demonstrates the effect of static electron correlation on SMAS within the DFT+U formalism. The nonmagnetic (non-spin-polarized) calculation results are shown in Supplementary Fig. 4 for two different values of U_{eff}=4, 8 eV. Comparing the PDOS without (see Fig. 3 in the main text) and with U, we find that the inclusion of U on Mn d orbitals pushes occupied d states downward, affecting the Mn d and Si p hybridization. In Supplementary Fig. 4a, occupied B_g and A_g peaks in the PDOS are located around −1 eV. As U is increased from 4 to 8 eV, B_g peaks are further pushed downward and are now located around −3 eV, lying lower in

energy than A_g^{out}.

Supplementary Fig. 5 and Supplementary Table 1 together present our initial magnetic configurations and the summary of DFT+U calculation results. The inclusion of the mean-field U value allows both the collinear ferromagnetic (FM) and antiferromagnetic (AF) solutions, but in the range of $0 < U \leq 4$ eV AF solution is lower in energy than the FM solution. Additional calculations by a parameter-free SCAN functional also yield the AF solution to be more stable than the FM one. DFT+U calculations with $U \geq 6$ eV does yield FM solution, but with the size of Mn moments to be $\sim 4\ \mu_B$, inconsistent with experimental observations. Hence, simple band calculations and static mean-field treatments of electron correlations cannot account for the observation of the subtle ferromagnetic instability.

Supplementary Note 6. Gap opening at Dirac cone and further band flattening via spin-orbit coupling

Despite the strength of SOC at the Mn site is weak, SOC still can affect electronic properties significantly when bands are nearly degenerate and bandwidth is small. Supplementary Fig. 6 summarizes the SOC effect on DMFT spectral function. The results are obtained for (U, J_H)=(4, 0.8) and (10, 0.8) eV at 116 K using Ising-type Coulomb interactions. Supplementary Fig. 6a shows the presence of the dispersive B_g-bands along the K−Γ direction with a bandwidth of ∼ 0.30 eV. Inclusion of SOC induces a small gap between Dirac point and flat band at K point as shown in Supplementary Fig. 6b. Because of admixture of other states in the presence of SOC, bandwidth of nodal surface band slightly enhances.

The most striking feature of the spectral function at $U = 10$ eV is the presence of a nearly flat band just below E_F, lying along the high symmetry path K−Γ. Without SOC, such a flat band state at the $k_z = 0$ plane was present below E_F (see Supplementary Fig. 6c). Including the SOC removes the Dirac points located close to E_F at K and opens up a gap of 10 meV, which makes the almost-flat band even flatter along the K−Γ direction. On the other hand, because of the weak strength of SOC, the $k_z = \pi$ nodal surface bands are almost unaffected. The SOC-induced band anticrossings at K may result in significant Berry-phase effects like spin-Hall responses.

The correlation-induced flat bands and their topological nature in the presence of SOC might make SMAS a promising candidate to see a signature of spin Hall or anomalous Hall conductivity, as reported in other kagome metals CoSn [16, 17] or YMn_6Sn_6 [18]. Further, we speculate that this system might exhibit negative magnetoresistance at low temperatures. It has been suggested

in the literature that the presence of flat bands close to the E_F induces ferromagnetic fluctuations that increase the scattering probability in a kagome plane. Indeed, our isothermal magnetization measurement at 2 K confirms this notion. At very low temperatures, an application of an external magnetic field might therefore suppress low-energy magnetic excitation to give rise to negative magnetoresistance [19, 20].

Supplementary Note 7. Exact and other choice of nominal occupancy in nominal double counting scheme

The choice of double-counting scheme affects DMFT results and conclusions. In the main text, we presented results with employing the nominal double-counting scheme [5] with the nominal charge of $n = 5.0$. Supplementary Figs. 7 and 8 summarize the results with $n = 4.5$ and 5.5, respectively. At $n = 4.5$ the flat band is pushed up to E_F and more strongly renormalized (Supplementary Fig. 7). On the other hand, as n is increased to 5.5, the flat band is pushed downward and overall feature of electron correlations is significantly weakened (Supplementary Fig. 8). At $n = 5.5$, the spectral function with $U = 4$ eV is very similar to the band dispersion calculated from simple DFT (see Fig. 3a-c in the main text for comparison).

Because the results critically depend on the choice of nominal charge, the results need to be tested with employing a parameter-free double-counting scheme. Hence, we checked our results using an exact double-counting scheme [21]. Supplementary Fig. 9 shows the spectral function with the exact double-counting scheme, with the choice of (U, J_H)=(8, 0.8) eV and full Coulomb interaction at $T = 300$ K. The result looks very similar to the one from the nominal double-counting scheme with $n = 5.0$ (see Fig. 4c in the main text), where the d-orbital occupancy converges to 5.34.

Supplementary Note 8. Orbital contribution of each atom to flat bands

The top and middle panels of Supplementary Fig. 10 show the orbital contribution of five Mn d orbitals, Sc d, Al and Si p orbitals from nonmagnetic LDA calculations. In addition, Supplementary Fig. 10 plots momentum and frequency dependent spectral function in the bottom panels for all five Mn d orbitals, obtained using same parameter settings as used in Fig. 3 of the main text. As evident from the plots, flat bands predominantly originate from the kagome lattice, formed by the Mn atoms. It also confirms that flat bands carry orbital character of d_{xz} and d_{yz}.

Supplementary Note 9. DFT+RISB mean-field calculation results

Employing $(U, J_{\mathrm{H}}) = (8, 0.8)$ eV and full Coulomb interactions, we checked that DMFT calculations with two different magnetic initial configurations (FM and AF, see Supplementary Fig. 5) do not admit any magnetism and converge into the paramagnetic solution above $T = 116$ K. This is in contrast to our DFT+U calculation results, which stabilize either antiferromagnetic order or ferromagnetism with large Mn moments, but consistent with experimental observations. Therefore, to make a direct check of ferromagnetic instabilities observed in our experimental results at low temperature, we employed rotationally-invariant Slave-boson (RISB) mean-field theory in combination with DFT (DFT+RISB). DFT+RISB method has been known to capture the correlation-induced band renormalization of the so-called coherent peak close to the Mott transition, and has been used to study electronic structures of various correlated metals at the zero-temperature limit [9, 10, 22]. To check whether our system is close to the ferromagnetic instability, we employed CYGUTZ package [23].

We observe the presence of the correlation-promoted flat bands close to the Fermi level with a proper choice of U and J_{H}, consistently with DMFT results (see Supplementary Fig. 11). The position of the kagome-flat B_g bands is strongly modified by the value of the J_{H} parameter (also moderately by U), and stronger J_{H} pushes the flat bands closer to the Fermi level (compare Supplementary Fig. 11a, b). The closest agreement with the DMFT bands with $(U, J_{\mathrm{H}}) = (8, 0.8)$ eV is achieved when $(U, J_{\mathrm{H}}) = (15, 1.5)$ eV was chosen in the DFT+RISB calculations (Supplementary Fig. 11b). Note that this difference between the DMFT and RISB Coulomb parameters is from the limitation of the DFT+RISB methodology, which cannot describe Mott-insulating phase and requires larger Coulomb parameters than realistic ones for systems close to the Mott transition [24] (this has been remedied in the recent 'ghost-RISB' method [25]). Nonmagnetic solution is almost always favored over magnetic ones, except when the flat bands are promoted up to the Fermi level by increasing J_{H} up to 1.8 eV. At $(U, J_{\mathrm{H}}) = (15, 1.8)$ eV, a spin splitting of about 0.15 eV occurs at the K-point (see Supplementary Fig. 11c).

Although no signature of ferromagnetic long-range order has been observed in our system, our RISB result shows an example that the flat bands being close to the Fermi level can induce ferromagnetic instabilities, in contrast to DFT+U results mentioned above, and that the flat bands are indeed located in the vicinity of the Fermi level.

Supplementary Table 1. **Stability of magnetic configurations as a function of U.** Total energies and Mn moment sizes of SMAS from DFT+U calculations with varying U value.

U (in eV)	E_{FM}-E_{AF} (eV/f.u.)	E_{NM}-E_{AF} (eV/f.u.)	m_{AF} (μ_B)	m_{FM} (μ_B)
1	0.0018	0.0033	0.412	0.133
2	0.1012	0.1216	1.297	0.515
4	0.0143	1.8416	3.102	3.213
6	-0.1382	N/A	3.711	3.742
8	-0.1764	N/A	4.074	4.050

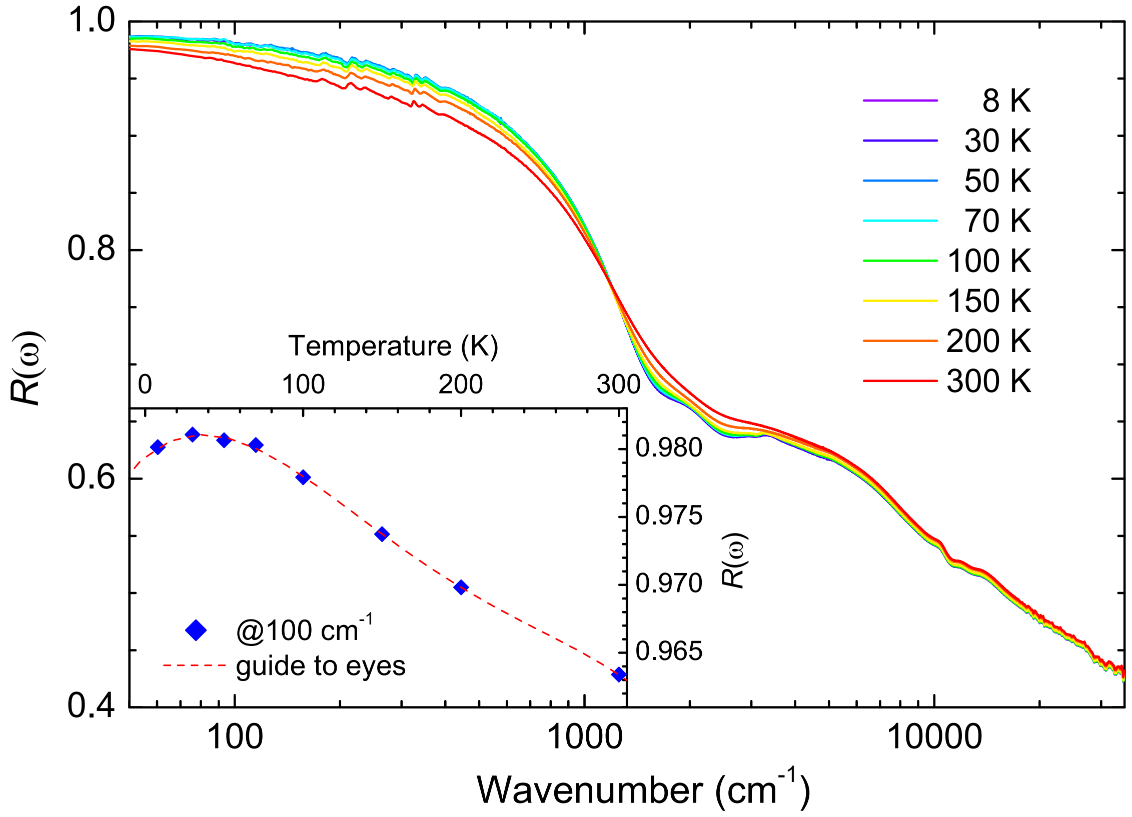

Supplementary Figure 1. **Reflectance spectra.** Measured reflectance spectra of SMAS at various selected temperatures. In the inset, the reflectance at 100 cm^{-1} is shown as a function of temperature.

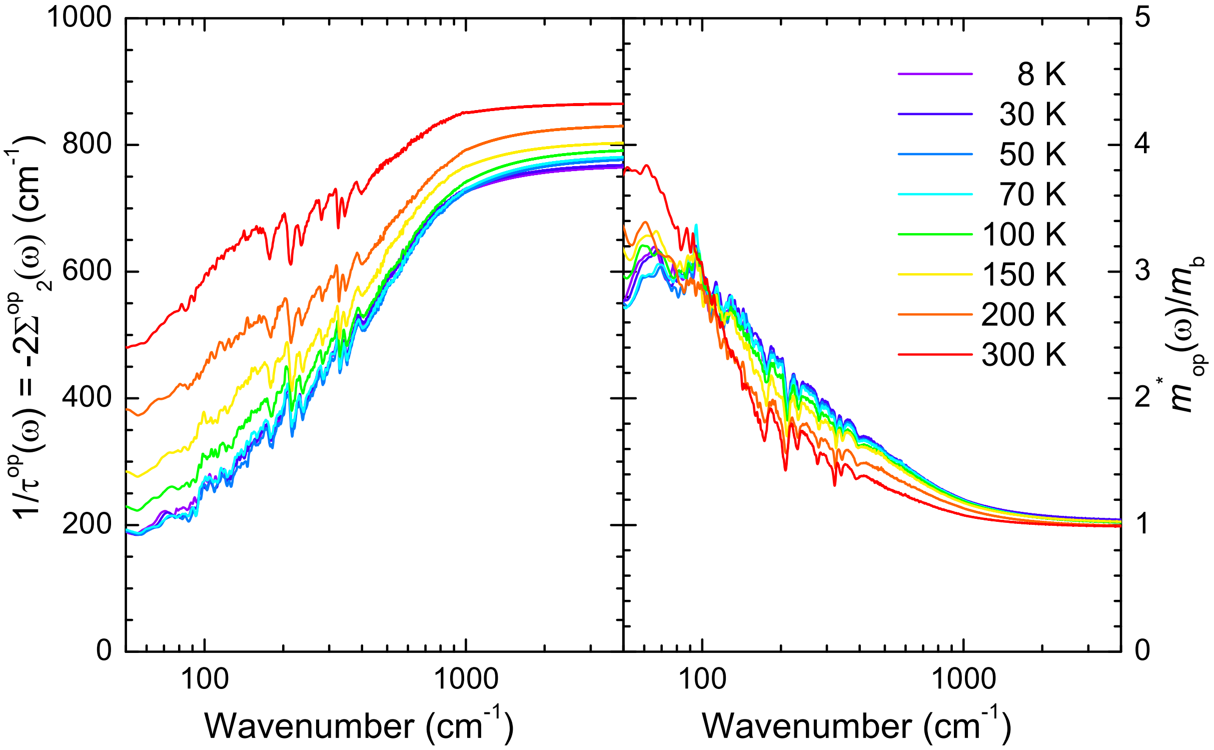

Supplementary Figure 2. **Optical scattering and effective mass.** Optical scattering rates and effective masses of SMAS at various temperatures.

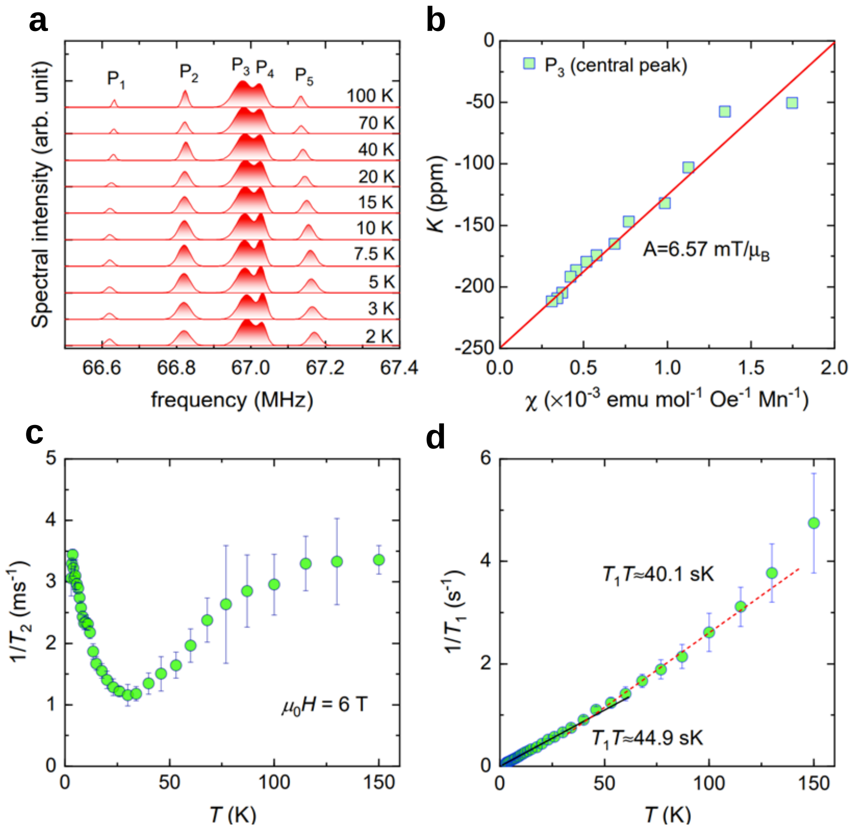

Supplementary Figure 3. ^{27}**Al NMR spectra. a** Temperature dependence of ^{27}Al NMR spectra of SMAS measured at $\mu_0 H = 6$ T. **b** Plot of the Knight shift vs the static magnetic susceptibility. The solid line is the fit to the Clogston-Jaccarino relation. Temperature dependence of **c** the nuclear spin-spin relaxation rate $1/T_2$ and the spin-lattice relaxation rate $1/T_1$. **d** The solid and dashed lines are fits to the Korringa relation T_1T=constant.

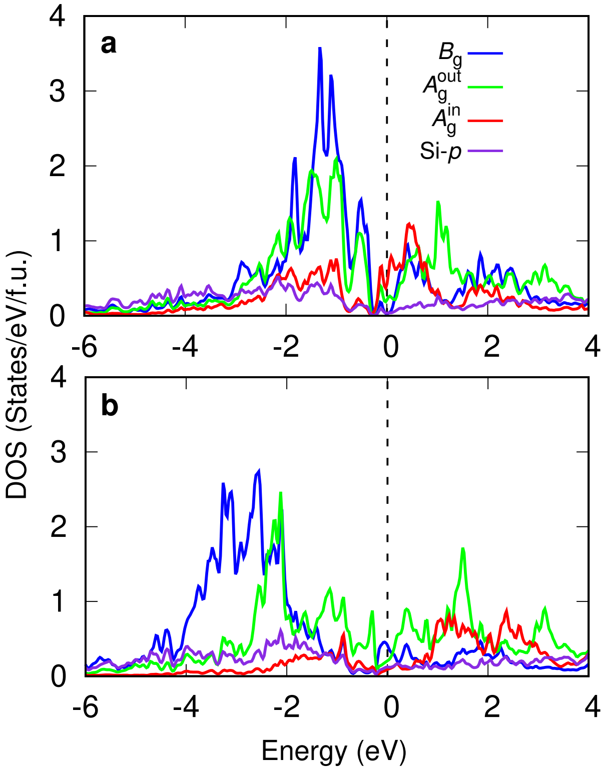

Supplementary Figure 4. **Effect of onsite Coulomb potential on SMAS. a**, **b** PDOS of SMAS from non-spin-polarized DFT+U calculations with Mn B_g (blue), A_g^{out} (green), A_g^{in} (red), and Si p (violet) orbitals for U=4, 8 eV, respectively.

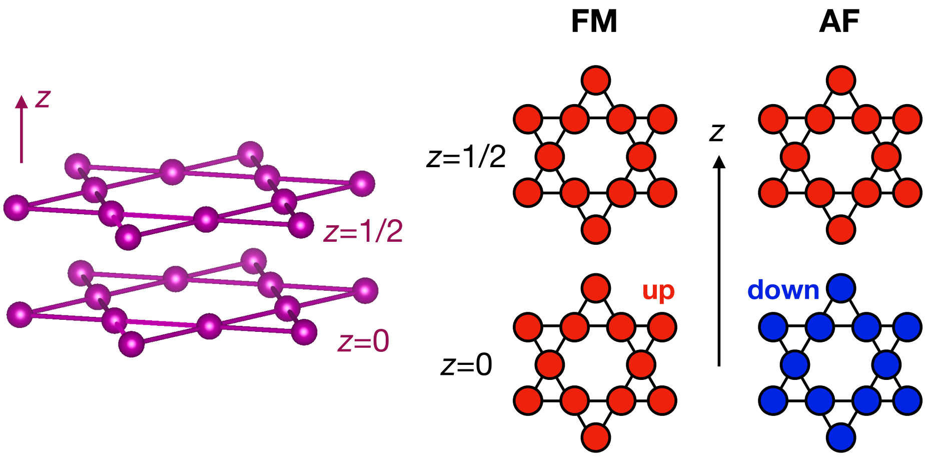

Supplementary Figure 5. **Schematic diagram of magnetic configurations.** Schematic illustrations of the FM and AF initial magnetic configurations we employed in DFT+U, DMFT, and slave-boson mean-field calculations.

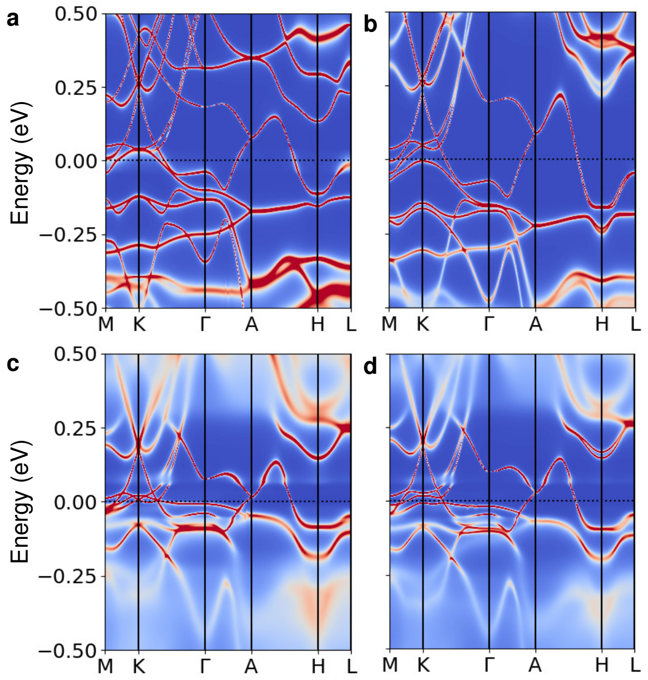

Supplementary Figure 6. **Effect of spin-orbit coupling on spectral function.** **a**, **b** Close-up view of spectral function without and with SOC for (U, J_H)=(4, 0.8) eV, and **c**, **d** (10, 0.8) eV at T=116 K using Ising-type Coulomb interaction, respectively.

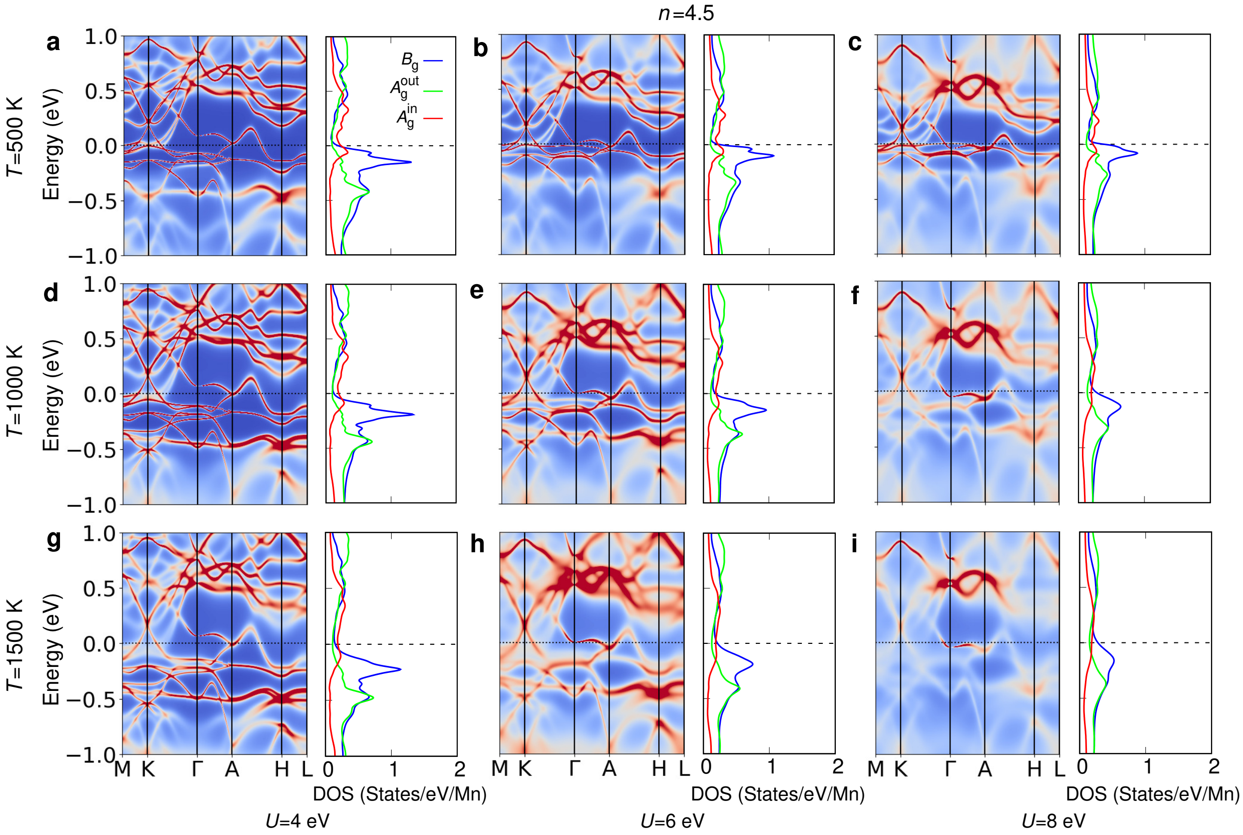

Supplementary Figure 7. ***U*, *T*, and double counting dependence on spectral function.** Spectral function and PDOS computed from DMFT, using full Coulomb interaction for three sets of (U, J_{H})=(4, 0.8), (6, 0.8), and (8, 0.8) eV and temperature T=500, 1000, and 1500 K for n = 4.5, respectively.

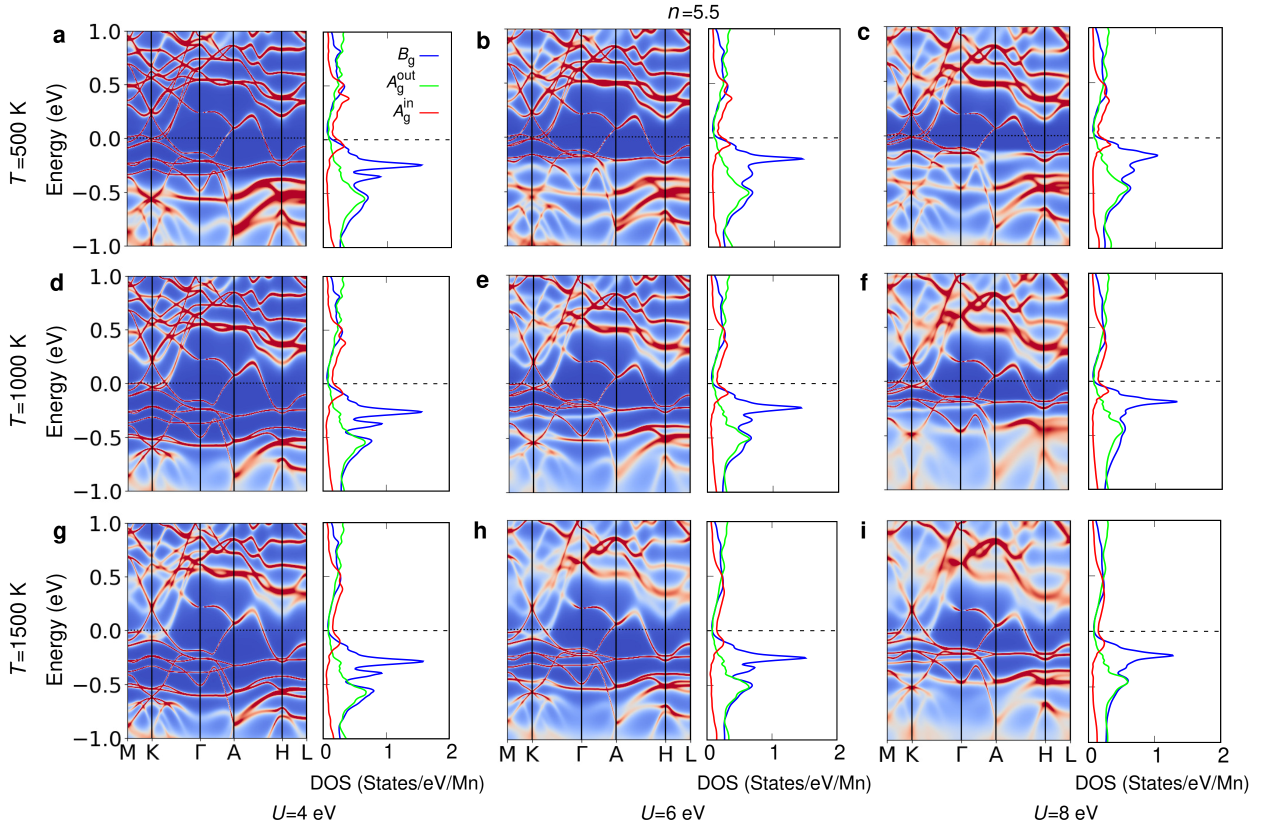

Supplementary Figure 8. ***U*, *T*, and double counting dependence on spectral function.** Spectral function alongside PDOS obtained from DMFT calculations, employing full Coulomb interaction for (U, J_{H})=(4, 0.8), (6, 0.8), and (8, 0.8) eV at T=500, 1000 and 1500 K, for n = 5.5, respectively.

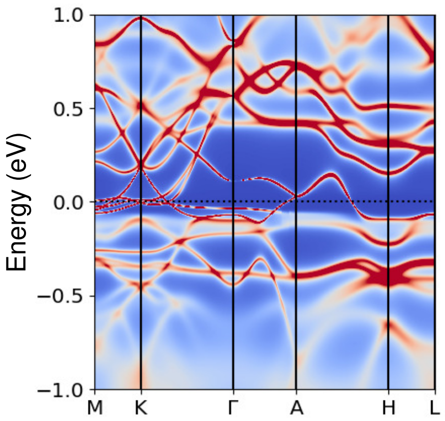

Supplementary Figure 9. **Spectral function from exact double counting scheme.** Spectral function of SMAS obtained, using exact double scheme and full Coulomb interaction for (U, J_H)=(8, 0.8) eV at T=300 K.

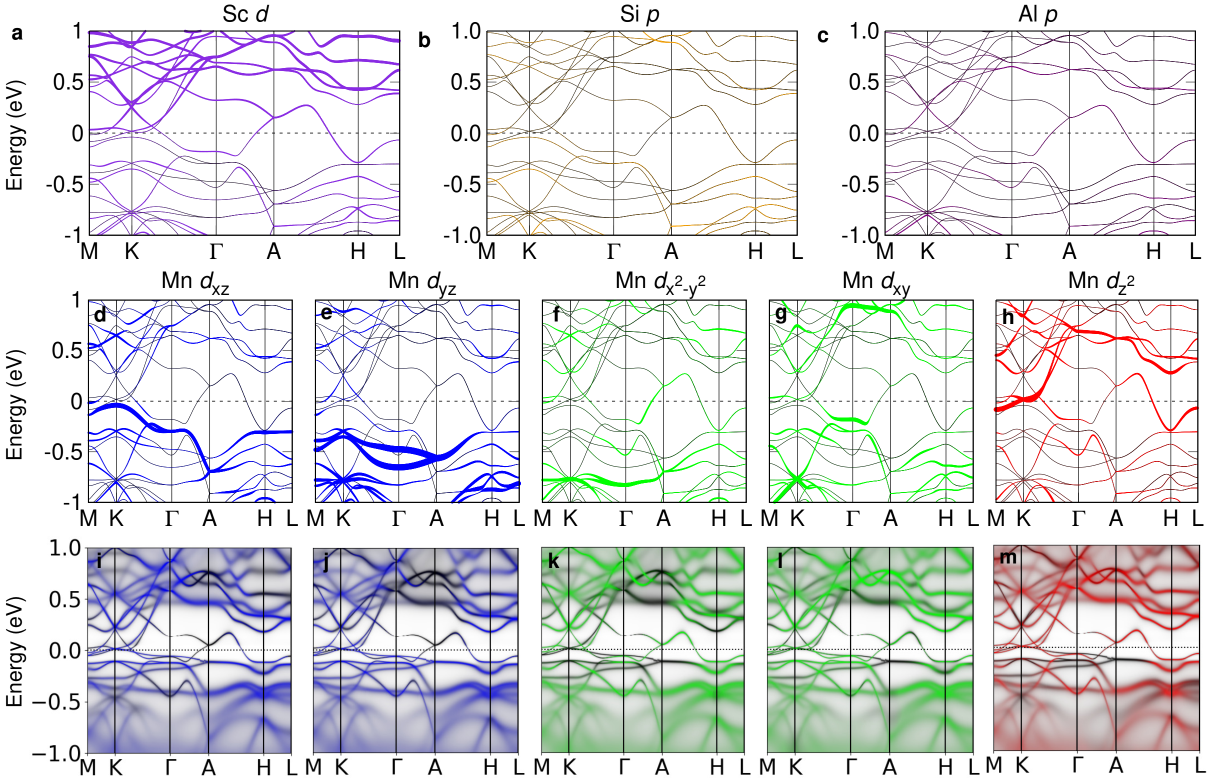

Supplementary Figure 10. **Orbital projected band structure and orbitally-resolved spectral function.** (Top and middle panels) Projected band structures display the orbital contribution of Mn, Sc -*d* and Si, Al -*p* orbitals from nonmagnetic DFT calculation. (Bottom panels) Momentum and frequency dependent spectral function from DMFT calculation for five Mn *d* orbitals is shown.

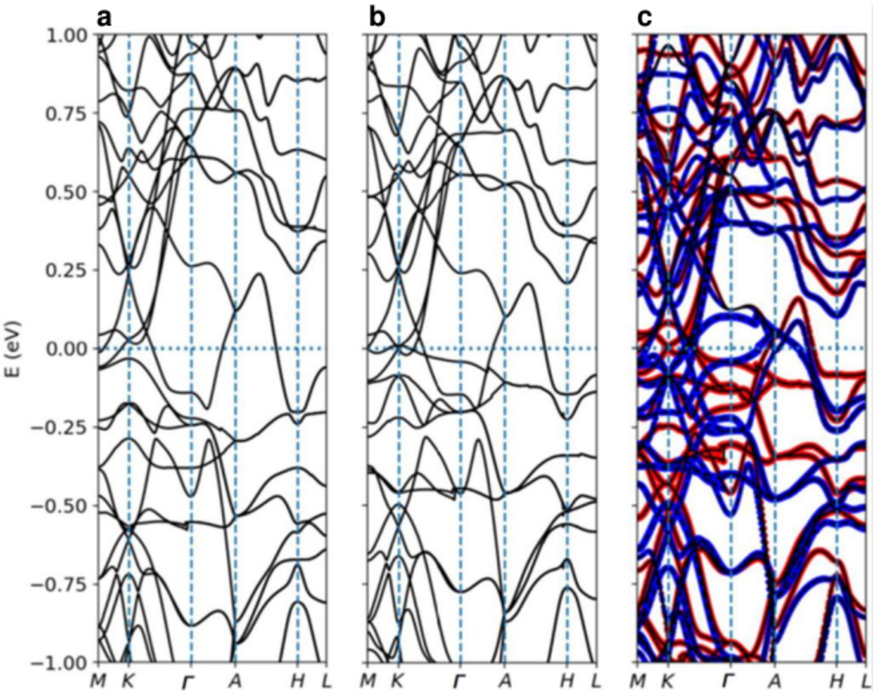

Supplementary Figure 11. **Band structure from DFT+RISB calculations.** Paramagnetic DFT+RISB bands with **a** $J_{\mathrm{H}} = 1.0$ and **b** 1.5 eV, where U is set to be 15 eV. Note that all the bands in the energy range are Mn d-orbital-originated. **c** Ferromagnetic solution at $(U, J_{\mathrm{H}}) = (15, 1.8)$ eV. Blue and red curves depict spin up and down bands, respectively.

Supplementary References

[1] C. C. Homes, M. Reedyk, D. A. Cradles, and T. Timusk, Technique for measuring the reflectance of irregular, submillimeter-sized samples, Appl. Opt. **32**, 2976 (1993).

[2] P. Blaha, K. Schwarz, G. K. H. Madsen, D. Kvasnicka, J. Luitz, R. Laskowski, F. Tran, and L. D. Marks, *WIEN2k: An Augmented Plane Wave plus Local Orbitals Program for Calculating Crystal Properties* (Vienna University of Technology, Austria, 2018).

[3] S. L. Dudarev, G. A. Botton, S. Y. Savrasov, C. J. Humphreys, and A. P. Sutton, Electron-energy-loss spectra and the structural stability of nickel oxide: An LSDA+U study, Phys. Rev. B **57**, 1505 (1998).

[4] J. P. Perdew, A. Ruzsinszky, G. I. Csonka, O. A. Vydrov, G. E. Scuseria, L. A. Constantin, X. Zhou, and K. Burke, Restoring the density-gradient expansion for exchange in solids and surfaces, Phys. Rev. Lett. **100**, 136406 (2008).

[5] K. Haule, C.-H. Yee, and K. Kim, Dynamical mean-field theory within the full-potential methods: Electronic structure of $CeIrIn_5$, $CeCoIn_5$, and $CeRhIn_5$, Phys. Rev. B **81**, 195107 (2010).

[6] K. Haule, Structural predictions for correlated electron materials using the functional dynamical mean field theory approach, J. Phys. Soc. Jpn. **87**, 041005 (2018).

[7] P. Werner, A. Comanac, L. de' Medici, M. Troyer, and A. J. Millis, Continuous-time solver for quantum impurity models, Phys. Rev. Lett. **97**, 076405 (2006).

[8] K. Haule, Quantum Monte Carlo impurity solver for cluster dynamical mean-field theory and electronic structure calculations with adjustable cluster base, Phys. Rev. B **75**, 155113 (2007).

[9] N. Lanatà, Y. Yao, C.-Z. Wang, K.-M. Ho, and G. Kotliar, Phase diagram and electronic structure of Praseodymium and Plutonium, Phys. Rev. X **5**, 011008 (2015).

[10] N. Lanatà, Y. Yao, X. Deng, V. Dobrosavljević, and G. Kotliar, Slave boson theory of orbital differentiation with crystal field effects: Application to UO_2, Phys. Rev. Lett. **118**, 126401 (2017).

[11] F. Wooten, *Optical Properties of Solids* (Academic, New York, 1972).

[12] D. B. Tanner, *Optical effects in solids* (Cambridge Univ. Press, 2019).

[13] W. Götze and P. Wölfle, Homogeneous dynamical conductivity of simple metals, Phys. Rev. B **6**, 1226 (1972).

[14] J. Hwang, T. Timusk, and G. D. Gu, High-transition-temperature superconductivity in the absence of the magnetic-resonance mode, Nature **427**, 714 (2004).

[15] J. Korringa, Nuclear magnetic relaxation and resonance line shift in metals, Physica **16**, 601 (1950).

[16] M. Kang, S. Fang, L. Ye, H. C. Po, J. Denlinger, C. Jozwiak, A. Bostwick, E. Rotenberg, E. Kaxiras, J. G. Checkelsky, and R. Comin, Topological flat bands in frustrated kagome lattice CoSn, Nat. Commun. **11**, 4004 (2020).

[17] Z. Liu, M. Li, Q. Wang, G. Wang, C. Wen, K. Jiang, X. Lu, S. Yan, Y. Huang, D. Shen, J.-X. Yin, Z. Wang, Z. Yin, H. Lei, and S. Wang, Orbital-selective Dirac fermions and extremely flat bands in frustrated kagome-lattice metal CoSn, Nat. Commun. **11**, 4002 (2020).

[18] M. Li, Q. Wang, G. Wang, Z. Yuan, W. Song, R. Lou, Z. Liu, Y. Huang, Z. Liu, H. Lei, Z. Yin, and S. Wang, Dirac cone, flat band and saddle point in kagome magnet YMn_6Sn_6, Nat. Commun. **12**, 3129 (2021).

[19] R. Arita, K. Kuroki, and H. Aoki, Electron-correlation-originated negative magnetoresistance in a system having a partly flat band, Phys. Rev. B **61**, 3207 (2000).

[20] J. Zhang, T. Yilmaz, J. W. R. Meier, J. Y. Pai, J. Lapano, H. X. Li, K. Kaznatcheev, E. Vescovo, A. Huon, M. Brahlek, T. Z. Ward, B. Lawrie, R. G. Moore, H. N. Lee, Y. L. Wang, H. Miao, and B. Sales, Flat band induced negative magnetoresistance in multi-orbital kagome metal (2021), arXiv:2105.08888 [cond-mat.str-el].

[21] K. Haule, Exact double counting in combining the dynamical mean field theory and the density functional theory, Phys. Rev. Lett. **115**, 196403 (2015).

[22] N. Lanatà, H. U. R. Strand, G. Giovannetti, B. Hellsing, L. de' Medici, and M. Capone, Orbital selectivity in Hund's metals: The iron chalcogenides, Phys. Rev. B **87**, 045122 (2013).

[23] Y. Yao, N. Lanatà, C.-Z. Wang, K.-M. Ho, and G. Kotliar, Gutzwiller variational embedding simulation package, CyGutz 10.6084/m9.figshare.11987439.v4 (2020).

[24] N. Lanatà, T.-H. Lee, Y.-X. Yao, V. Stevanović, and V. Dobrosavljević, Connection between mott physics and crystal structure in a series of transition metal binary compounds, npj Computational Materials **5**, 30 (2019).

[25] T.-H. Lee, C. Melnick, R. Adler, N. Lanatà, and G. Kotliar, Accuracy of ghost-rotationally-invariant slave-boson theory for multiorbital Hubbard models and realistic materials, Phys. Rev. B **108**, 245147 (2023).
